# Maternal healthcare quality, continuity, and experiences among pregnant adolescents and adult women in Ethiopia, Kenya, and South Africa: A cohort study

**DOI:** 10.1371/journal.pgph.0004729

**Published:** 2026-10-05

**Authors:** Beatrice Amboko, Irene Mugenya, Emily Odipo, Rose Jepchumba Kosgei, Wen-Chien Yang, Shalom Sabwa, Gebeyaw Molla Wondim, Nompumelelo Gloria Mfeka-Nkabinde, Londiwe Mthethwa, Nokuzola Cynthia Mzolo, Margaret E. Kruk, Catherine Arsenault, Jacinta Nzinga

**Affiliations:** 1 Health Economics Research Unit, KEMRI-Wellcome Trust Research Programme, Nairobi, Kenya; 2 Nuffield Department of Medicine, University of Oxford, Oxford, United Kingdom; 3 Population and Health Impact Surveillance Group, KEMRI-Wellcome Trust Research Programme, Nairobi, Kenya; 4 Department of Obstetrics and Gynaecology, Faculty of Health Sciences, University of Nairobi, Nairobi, Kenya; 5 Department of Global Health, Milken Institute School of Public Health, The George Washington University, Washington, District of Columbia, United States of America; 6 Department of Global Health and Population, Harvard T. H. Chan School of Public Health, Boston, Massachusetts, United States of America; 7 Department of Epidemiology, Rollins School of Public Health, Emory University, Atlanta, Georgia, United States of America; 8 Ethiopian Public Health Institute, Addis Ababa, Ethiopia; 9 Department of Family Medicine, Howard College Campus, University of KwaZulu-Natal, Durban, South Africa; 10 School of Nursing and Public Health, University of KwaZulu-Natal, Durban, South Africa; 11 School of Medicine, Washington University in St. Louis, St. Louis, Missouri, United States of America; 12 Liverpool School of Tropical Medicine, Liverpool, United Kingdom; Yale University, UNITED STATES OF AMERICA

## Abstract

The capacity of health systems to provide quality care for pregnant adolescents remains underexplored. This study examined the quality, continuity, and experiences of maternal healthcare among pregnant adolescents (<20 years) compared with adult women (20 + years) in Ethiopia, Kenya, and South Africa. It also explored factors associated with mistreatment and obstetric complications among adolescents using data from the Maternal and Newborn Health eCohort study. We followed 3,051 pregnant women from their first antenatal care (ANC) visit until six weeks postpartum. We used descriptive analyses to compare care quality and outcomes and logistic regression to identify factors among adolescents. The study included 380 adolescents and 2,671 adults. Adolescents had lower first-trimester ANC attendance (22% vs. 30%, p = 0.003), fewer follow-up ANC visits (mean 3.1 vs. 3.8, p < 0.001), and lower adherence to iron-folic acid supplementation (59.0% vs. 69.4%, p < 0.001). Continuity of care was also lower (53% vs. 59%, p < 0.001). Adolescents experienced more mistreatment during delivery (26.9% vs. 18.6%, p = 0.003). Obstetric complications occurred in 31% of adolescents compared with 25.9% of adults. Mistreatment was associated with rural residence (adjusted odds ratio (aOR): 2.61, 95% CI: 1.11–6.13, p = 0.028), public facility use (aOR: 4.33, 95% CI: 1.44–13.1, p = 0.009) and intimate partner violence (IPV) (aOR: 3.31, 95% CI: 1.16–9.39, p = 0.025). Protective factors included being in Kenya (aOR: 0.19, 95% CI: 0.05–0.75, p = 0.018), privacy during delivery (aOR: 0.44, 95% CI: 0.21–0.92, p = 0.030) and high perceptions of quality of care (aOR: 0.37, 95% CI: 0.18–0.76, p = 0.007). Obstetric complications were associated with rural residence (OR: 2.25, 95% CI: 1.09–4.63; p = 0.028) and antenatal depression (OR: 2.43, 95% CI: 1.19–5.00, p = 0.015). These findings highlight gaps in adolescent maternal healthcare. Strengthening adolescent-responsive and respectful care within primary healthcare, alongside integrated mental health and IPV support, may improve adolescents’ maternal healthcare experiences and outcomes.

## Introduction

Adolescent pregnancy is a global issue, causing physical, psychological and socioeconomic challenges for young mothers, newborns and their communities [1,2]. Adolescence is the period between 10 and 19 years of age [3]. This is a vulnerable period marked by the transition to adulthood and significant physical, psychological, and emotional changes [3,4]. Each year, an estimated 21 million pregnancies occur among adolescents in low and middle-income countries with the majority in sub-Saharan Africa (SSA) [2,5,6]. About a half of these pregnancies are unintended [2]. In SSA, pregnancy disproportionately affects adolescents from socio-economically disadvantaged backgrounds and with lower levels of education [7,8]. Compared to adult women, pregnant adolescents face greater biological risks due to physical prematurity. This can increase the risk of complications such as pre-eclampsia, anaemia, postpartum haemorrhage and obstructed labour [2,5,9]. In addition, adolescents experience heightened psychological vulnerability increasing their risk of postpartum depression and social challenges, such as stigma, limited autonomy and poor access to care [2].

Furthermore, infants born to adolescent mothers face an elevated risk of complications or death, including stillbirth, low birth weight, and premature births [10,11]. Psychosocial and economic consequences, including school dropout, social stigma, early marriage, and increased vulnerability to abuse, are also well documented [12,13]. These risks are exacerbated by age-related dependency, unequal power dynamics, and delays in seeking maternal healthcare services [14,15].

Health systems continue to support adolescents in delaying pregnancy [16]. However, when adolescents do become pregnant and choose to carry the pregnancy to term, they must be ensured access to high-quality, respectful and adolescent-responsive maternal health services [8]. Previous studies have shown that adolescents are less likely to attend the recommended number of antenatal care (ANC) visits and to deliver in health facilities [17,18]. Even when they do access care, they may receive lower quality services and less social support than adult women [19]. This underscore persistent gaps in access to and quality of maternal and neonatal health services for adolescents in SSA.

Previous studies in SSA have also documented widespread mistreatment and poor delivery outcomes among adolescents. A cross-sectional study in Kenya found that adolescents experienced higher levels of mistreatment during childbirth, including verbal abuse, neglect, and denial of care, which were associated with lower satisfaction [20]. Obstetric complications in this group have been linked to delayed ANC initiation, poor service quality and continuity, limited decision-making autonomy, and socio-economic disadvantage [21,22].

A recent scoping review also highlighted that pregnant and parenting adolescents in SSA frequently face stigma, social exclusion, and judgmental attitudes from healthcare providers and communities. These experiences are often compounded by a lack of supportive services tailored to their unique needs [23]. However, much of the existing literature is either country specific, not adolescent focused, or based on cross-sectional data.

To address this gap, this study used longitudinal multicountry data from Ethiopia, Kenya, and South Africa to compare the quality, continuity, and experience of maternal healthcare among adolescents and adult women. The longitudinal design allowed us to follow women from their first ANC visit through delivery and the postnatal period, providing a comprehensive assessment of care across the maternal healthcare continuum. The study also identifies key risk factors for mistreatment and obstetric complications. By combining clinical and experiential quality measures, the study provides a more comprehensive understanding of adolescent-specific gaps in maternal healthcare. These findings generate important insights to guide adolescent-responsive health system improvements across SSA.

## Study methods

### Ethics statement

The study protocol was reviewed and approved by the Institutional Review Boards (IRB) of the Harvard T.H. Chan School of Public Health (protocol #IRB22–0487), the Kenya Medical Research Institute (protocol number KEMRI/SERU/CGMR-C/4226), the Ethiopian Public Health Institute (protocol number EPHI-IRB-448–2022) and the University of KwaZulu-Natal in South Africa (protocol number BREC/00004645/2022). Formal written informed consent was obtained from all adult study participants or emancipated minors or from formal guardians or next-of-kins for study participants under 18 years old per local regulations. A data-sharing agreement was established across countries. Data were de-identified, securely transferred via encrypted platforms, and stored using password-protected systems with unique study IDs to protect participant privacy.

### Data source

This analysis used data from the Maternal and Newborn Health (MNH) eCohort in Ethiopia, Kenya and South Africa. This was a longitudinal health system quality study that enrolled women face-to-face during their first ANC visit and followed them by phone through pregnancy, delivery, and the postnatal period. The main exposure of interest was age group, comparing adolescents aged 15–19 to adults aged 20 years and above.

Participants were recruited consecutively from public and private health facilities in Ethiopia and Kenya, and from public facilities in South Africa. Women were eligible if they were pregnant, aged 15 or older, attending their first ANC visit, and planned to continue receiving care in the same area. Women were excluded if they had already received ANC from a skilled healthcare provider, visited only to confirm the pregnancy, or were under 15 years old.

Data collection included a baseline interview at enrolment after the first ANC visit and follow-up phone interviews throughout the maternal healthcare continuum. Detailed methods and sample size calculations are published elsewhere [24–26]. For this analysis, we defined adolescents as those aged 15–19 years and adults as those aged 20 years and above, based on the WHO definition of adolescence (10–19 years) [3] and the cohort’s eligibility criteria.

Data were collected through three structured questionnaire modules: module 1 included a baseline face-to-face interview at the first ANC visit; module 2 was a monthly follow-up phone interview during the pre-natal period; and module 3 was a post-delivery phone interview conducted around 2 to six weeks postpartum. Information was collected on demographic characteristics, content of care (assessments, diagnostic tests, treatments, counselling), delivery outcomes and patient perceived quality of care. Data were collected between 3^rd^ April 2023 and 28^th^ February 2024 in Ethiopia, 12^th^ June 2023 and 3^rd^ July 2024 in Kenya, and 24^th^ April 2023 and 24^th^ July 2024 in South Africa. In this manuscript, we present cumulative data from adolescent girls aged 15–19 years and adult women aged 20 years and above, collected from enrolment through six weeks post-delivery.

### Measures

#### Quality and continuity of care.

Quality of care was defined using both clinical measures such as the physical assessments, diagnostic tests and treatments provided, to assess adherence to evidence-based guidelines [27–32], and patient-reported experiences. Indicators were organized along the maternal healthcare continuum, the antenatal period, delivery care, and postnatal care, and assessed through both the content of services received and participants’ perceptions of care. These indicators are detailed in Box 1 and were used to quantify both the quality and continuity of care across service points.

To understand the quality of care provided, we assessed both the content of care and the participant’s perceptions of the services received during ANC, delivery care and the early postnatal period. Specifically, we examined the assessments, tests and treatments they received, including the recommended supplements during pregnancy. To assess continuity of care, we calculated a continuity of care index based on the proportion of women who met four key criteria: (1) attended their first ANC visit during the first trimester, (2) completed four or more ANC visits during pregnancy, (3) delivered at a health facility, and (4) received a postnatal check-up before discharge.

**Box 1. Measures of quality and continuity of care across the maternal healthcare continuum.**

**Antenatal care**

**First ANC visit**

1. First ANC attendance in the first trimester
2. First ANC screening and treatment completeness index^a^
3. First ANC counselling completeness index^b^
4. Overall rating of the quality of first ANC visits as very good/excellent^c^

**Follow-up (FU) ANC visits**

1. Proportion with at least one blood test after the first ANC visit
2. Proportion with at least one urine test after the first ANC visit
3. Proportion who received food supplementation at least once during the antenatal period
4. Proportion who had ultrasound done at one point (baseline or FU ANC visits)
5. Adherence to iron and folic acid supplementation (IFAS)^d^
6. Proportion who were counselled on postpartum family planning at least once
7. Total number of ANC visits

**Delivery care**

1. Proportion who delivered at a health facility
2. Consent and privacy for vaginal examinations (VE)
3. Appropriate pain relief^e^
4. Proportion who had adequate privacy during delivery using curtains, partitions or other measures
5. Proportion who rated the overall quality of delivery care as very good/excellent
6. Likelihood of recommending the health care provider to others for childbirth^f^

**Immediate postnatal care**

1. Proportion who had a postnatal check-up before discharge
2. Immediate postnatal counselling index^g^

**Continuity of care**

1. A continuity of care index calculated from the proportion who had:
a. their first ANC visit during the first trimester,
b. four or more ANC visits during the entire pregnancy,
c. delivered at a health facility
d. received a postnatal check-up before discharge

^a^ First ANC visit screening and treatment index included: measurements of blood pressure, weight, height, and mid-upper arm circumference (MUAC), as well as tests for HIV, syphilis, blood sugar, urine, and haemoglobin levels, iron and folic acid supplementation (IFAS) and tetanus injection across all countries and calcium supplementation in Ethiopia and South Africa. Calcium supplementation was not included in the index for Kenya, reflecting differences in national ANC guidelines, as it is not required as part of routine ANC in Kenya [29].

^b^ First ANC visit counselling index included being counselled on danger signs during pregnancy, nutrition, physical exercise, birth plan and scheduling of follow-up visits.

^c^ Women were asked to rate the overall quality of their first ANC visit on a 5-point Likert scale from “very poor” to “excellent”.

^d^ Women who continued to take IFAS at least once during follow-up ANC.

^e^ Included requesting or was given or received pain relief during delivery.

^f^ Women were asked to rate whether they were likely to recommend their birth facility or provider to a family member or friend for childbirth on a 4-point Likert scale from “not at all likely” to “very likely”.

^g^ Immediate postnatal counselling index included being counselled on what the baby should eat, umbilical cord care, keeping the baby warm, returning for baby vaccinations, hand washing with soap, danger signs for the baby and danger signs for the mother before discharge after delivery.

#### Risk factors for adverse delivery outcomes (mistreatment during childbirth and obstetric complications) among adolescents.

We assessed factors associated with mistreatment during childbirth and adverse delivery outcomes among adolescents only, given their increased risk of both. In this study, mistreatment is treated as a dimension of quality of care, specifically the experience of care. Mistreatment was assessed using eight questions on physical and verbal abuse, with four questions for each domain, following recommendations by Leslie et al. [33] (see S1 Table).

To examine obstetric complications, we analysed key adverse delivery outcomes women may encounter during delivery and the immediate postpartum period. These outcomes included prolonged labour exceeding 12 hours, admission to the intensive care unit (ICU), symptoms of obstetric fistula, the need for blood transfusions, and self-reported severe delivery complications [34].

Lastly, we examined associations between a range of potential predictive factors and adverse delivery outcomes. We explored geographical and delivery facility characteristics, sociodemographic factors, reproductive and obstetric characteristics, and medical and obstetric history as well as ANC content and user experience as outlined in Box 2.

**Box 2. List of assessed factors.**

A) **Geographical factors**

1. Country of residence

2. Region of residence

B) **Delivery facility characteristics**

1. Facility level

2. Facility ownership

3. Visited another facility before delivery facility

C) **Sociodemographic characteristics**

1. Age,

2. Marital status

3. Education level

4. Wealth tertiles^a^

5. Health literacy level^b^

6. Employment status

D) **Reproductive and obstetric factors**

1. Gravidity

2. Gestational age at the first ANC visit

3. Pregnancy intentionality

E) **Medical or obstetric history factors**

1. Experienced at least one pregnancy danger sign,

2. Prenatal depression (PHQ-9 > 5)^c^

3. Engaged in risky health behaviour like substance abuse,

D) **Experienced of intimate partner violence (IPV)**^d^

E) **ANC content of care**

F) **User experience**

1. Overall rating of the quality of first ANC visit

2. Overall rating of the quality of delivery care

^a^ Wealth tertiles were based on a country-specific wealth index derived from principal components analysis of binary housing and asset ownership indicators, following the Demographic and Health survey approach. Indicators included improved water source, sanitation, cooking fuel, floor, wall and roof, electricity, and ownership of a radio, television, telephone, refrigerator, bicycle, motorcycle, car and bank account. The first component was used to group participants into within-country tertiles.

^b^ Health literacy was assessed using six health knowledge questions adapted from the Indian Health and Human Development Survey [35] (see S2 Table). We presented the results as the percentage of women who correctly answered all the six questions.

^c^ Symptoms of prenatal depression were assessed using the nine-item Patient Health Questionnaire (PHQ-9), with a cut-off score of >5 to capture mild to severe depressive symptoms.

^d^ Assessed through self-reported physical or emotional/verbal abuse by a current or former intimate partner based on Demographic and Health survey approach.

### Statistical analysis

Our primary focus was to compare the key measures between adolescents and adults within each country. Descriptive analyses were stratified by the two age groups. We used chi-square tests and t-tests to assess differences in the quality of care and outcomes between adolescents and adults. To assess the factors associated with adverse delivery outcomes, we used logistic regression for the subsample of adolescents only across the three countries. These analyses combined adolescent girls from all three countries and included countries as fixed effects. We undertook multivariable regression analyses. The results are presented as adjusted odds ratio (aOR) with 95% confidence intervals (CI). A p-value of <0.05 was considered statistically significant.

Although the MNH eCohort study was longitudinal, this analysis used cumulative data collected from enrolment at the first ANC visit through six weeks postpartum. Specifically, baseline data (module 1) were used to assess participant characteristics and the quality of the first ANC visit. The quality of follow-up ANC care (module 2) and delivery and immediate postnatal care (module 3) were assessed using data collected through phone surveys during pregnancy and after delivery. Data from these repeated monthly interviews were aggregated to create summary indicators for each stage of care. All analyses were conducted using Stata version 17 (StataCorp, College Station, TX, USA).

### Inclusivity in global research

Additional information regarding the ethical, cultural, and scientific considerations specific to inclusivity in global research is included in the Supporting Information (see S1 Checklist).

## Results

### Sociodemographic characteristics

Table 1 summarises the demographic and health characteristics of 3,051 women included in the study across Ethiopia, Kenya, and South Africa. The proportion of adolescents ranged from 9% to 17% across the three countries, with a mean age of 18. In contrast, adult women had a mean age of 28 years. Among adolescents, 13% to 24% were aged 15–17, while 76% to 88% were aged 18–19. Within each country, adolescents were generally poorer, less educated, more likely to be unemployed and to reside in rural areas. Marital status varied significantly by country with most adolescents married in Ethiopia but unmarried in Kenya and South Africa.

**Table 1:** Characteristics of participants in the maternal and newborn health (MNH) eCohort study in Ethiopia, Kenya and South Africa, 2023-2024.

	Ethiopia		Kenya		South Africa		Total		
	Adolescent (N = 86, 8.6%)	Adults (N = 914, 91.4%)	Adolescents (N = 121, 12.0%)	Adults (N = 888, 88.0%)	Adolescents (N = 173, 16.6%)	Adults (N = 869, 83.4%)	Adolescents (N = 380, 12.5%)	Adults (N = 2,671, 87.5%)	Total (N = 3,051)
**Age, mean (SD)** ^**a**^	18.2 (0.9)	26.1 (4.5)	18.0 (1.0)	28.0 (5.7)	18.0 (0.97)	28.4 (5.71)	18.1 (0.9)	27.5 (5.4)	26.3 (6.0)
**Marital status:** Married or partnered, n (%)	82 (95.4)	890 (97.5)	57 (47.5)	730 (82.5)	2 (1.2)	124 (14.3)	141 (37.2)	1744 (65.4)	1885 (61.9)
**Education level**, n (%)									
None or some primary education	54 (62.8)	330 (36.1)	8 (6.6)	62 (7.0)	1 (0.6)	10 (1.2)	63 (16.6)	402 (15.1)	465 (15.3)
Completed primary education	31 (36.0)	364 (39.9)	71 (58.7)	280 (31.5)	89 (51.5)	206 (23.7)	191 (50.3)	850 (31.9)	1041 (34.1)
Completed secondary or higher	1 (1.2)	219 (24.0)	42 (34.7)	546 (61.5)	83 (48.0)	652 (75.2)	126 (33.2)	1417 (53.1)	1543 (50.6)
**Wealth tertiles** ^**b**^**, n (%)**									
Poorest	32 (37.6)	166 (18.5)	48 (39.7)	154 (17.5)	40 (23.1)	224 (25.8)	120 (31.7)	544 (20.6)	664 (22.0)
Middle	48 (56.5)	166 (18.5)	63 (52.1)	545 (61.8)	72 (41.6)	338 (38.9)	183 (48.3)	1423 (53.8)	1606 (53.1)
Richest	5 (5.9)	540 (60.3)	10 (8.3)	183 (20.8)	61 (35.3)	306 (35.3)	76 (20.1)	679 (25.7)	755 (25.0)
**Region**, n (%)									
Rural	60 (69.8)	448 (49.0)	89 (73.6)	418 (47.0)	115 (66.5)	400 (46.0)	264 (69.5)	1265 (47.4)	1529 (50.1)
**Very good health literacy level** ^**c**^, n (%)	5 (5.8)	261 (28.6)	35 (28.9)	416 (46.8)	35 (20.2)	310 (35.7)	75 (19.7)	987 (37.0)	1062 (34.8)
**Employment status**, n (%)									
Employed	16 (18.6)	371 (40.6)	24 (20.2)	522 (58.8)	0	193 (22.2)	40 (10.6)	1086 (40.7)	1126 (37.0)
**Social support, mean (SD)**	3.3 (2.4)	3.0 (2.2)	3.3 (2.1)	3.2 (2.2)	2.5 (1.5)	2.6 (1.8)	3.0 (2.0)	3.0 (2.1)	3.0 (2.1)
**Gravidity mean (SD)**	1.2 (0.5)	2.6 (1.4)	1.2 (0.6)	2.7 (1.5)	1.1 (0.3)	2.5 (1.3)	1.2 (0.5)	2.6 (1.4)	2.4 (1.4)
**Gestational age at first ANC** ^**d**^ **visit**, n (%)									
1st trimester 0–12 weeks	18 (21.4)	286 (31.9)	9 (7.4)	147 (16.6)	59 (34.3)	366 (42.2)	86 (22.8)	799 (30.1)	885 (29.2)
2nd trimester 13–26 weeks	60 (71.4)	555 (61.9)	74 (61.2)	564 (63.5)	89 (51.7)	418 (48.2)	223 (59.2)	1537 (58.0)	1760 (58.1)
3rd trimester 27–42 weeks	6 (7.1)	55 (6.1)	38 (31.4)	177 (19.9)	24 (14.0)	84 (9.7)	68 (18.0)	316 (11.9)	384 (12.7)
**Pregnancy was intended,** n (%)	61 (70.9)	667 (73.1)	48 (40.0)	567 (64.7)	9 (5.2)	172 (19.8)	118 (31.2)	1405 (52.9)	1523 (52.9)
**Current medical/obstetric history**, n (%)									
Underweight (MUAC ^e^ < 23 cm or BMI ^f^ < 18.5)	33 (38.4)	239 (26.2)	9 (7.4)	37 (4.2)	5 (2.9)	12 (1.4)	47 (12.4)	288 (10.8)	335 (11.0)
Experienced at least one pregnancy danger sign ^g^	21 (24.4)	221 (24.2)	25 (20.7)	208 (23.4)	82 (47.7)	400 (46.4)	128 (33.8)	829 (31.1)	957 (31.4)
Experienced mild to severe ANC depression (PHQ9 > 5 ^h^)	16 (18.6)	231 (25.3)	21 (17.4)	179 (20.2)	34 (20.0)	116 (13.4)	71 (18.8)	526 (19.7)	597 (19.6)
Rates own health as poor or fair	10 (11.6)	160 (17.5)	7 (5.8)	80 (9.0)	18 (10.4)	64 (7.4)	35 (9.2)	304 (11.4)	339 (11.1)
Engaged in risky health behaviour (substance use)	15 (17.4)	224 (24.6)	3 (2.5)	14 (1.6)	11 (6.4)	82 (9.4)	29 (7.6)	320 (12.0)	349 (11.5)
**Experienced Intimate Partner Violence** (emotional/physical), n (%)	7 (8.1)	58 (6.3)	10 (8.3)	60 (6.7)	18 (10.4)	48 (5.5)	35 (9.2)	166 (6.2)	201 (6.6)

^a^SD: Standard deviation; ^b^ Was constructed using principal component analysis using questions about household assets and the materials used to construct the home; ^c^ The percentage of women who correctly answered six questions designed to assess their health knowledge; ^d^ ANC: Antenatal care; ^e^ MUAC: Mid-upper arm circumference: ^f^ BMI: Body Mass Index; ^g^ Experiencing at least one of the following: persistent or severe headaches, any vaginal bleeding, fever, intense abdominal pain (beyond general discomfort), convulsions or seizures, repeated episodes of fainting, or loss of consciousness; ^h^ PHQ9: Patient Health Questionnaire-9 [36]

Health literacy was lower among adolescents than adults (20% vs. 37%), and fewer initiated ANC in the first trimester (23% vs. 30%). Most adolescents were in their first pregnancy. A third of adolescents reported at least one danger sign during pregnancy, while 9% reported IPV (Table 1).

### Quality and continuity of maternal health care

Table 2 shows the quality of antenatal, delivery, and postnatal care, while Fig 1 illustrates the proportion of women receiving care along the continuum of maternal healthcare across the three countries.

**Table 2:** Quality of maternal health care received by adolescents compared to adult women among women enrolled in the MNH eCohort study in Ethiopia, Kenya and South Africa, 2023-2024.

	Ethiopia			Kenya			South Africa			Total		
	Adolescents	Adults	P-value	Adolescents	Adults	P-value	Adolescents	Adults	P-value	Adolescents	Adults	P-value
ANTENATAL CARE	N = 86	N = 914		N = 121	N = 888		N = 173	N = 876		N = 380	N = 2,671	
**First ANC visit**												
First ANC completeness index: screening and treatments (mean (SD))	49.4 (14.9)	50.9 (15.5)	0.367	70.9 (15.1)	69.3 (14.5)	0.270	85.4 (7.9)	84.6 (8.7)	0.644	72.6 (18.6)	68.0 (19.2)	**<0.001**
First ANC completeness index: counselling (mean (SD))	31.3 (18.9)	33.7 (20.7)	0.280	50.3 (30.0)	60.6 (29.6)	**<0.001**	56.7 (27.6)	57.8 (27.8)	0.865	48.9 (28.5)	50.5 (28.9)	0.310
**Follow-up (FU) ANC visits**												
Number of additional ANC visits (mean, SD)	2.6 (1.7)	3.1 (1.9)	**0.033**	1.8 (1.5)	2.1 (1.8)	**0.042**	1.8 (2.0)	2.2 (2.3)	0.406	2.0 (1.8)	2.5 (2.1)	**<0.001**
Total number of ANC visits (mean, SD)	3.9 (1.8)	4.7 (2.2)	**0.002**	2.9 (1.6)	3.3 (1.9)	**0.032**	2.8 (2.0)	3.2 (2.3)	0.406	3.1 (1.9)	3.8 (2.3)	**<0.001**
At least one blood test during FU ANC (n (%))	34 (39.5)	446 (48.8)	0.100	43 (35.5)	315 (35.5)	0.989	93 (53.8)	523 (60.2)	0.116	170 (44.7)	1,284 (48.1)	0.223
At least one urine test during FU ANC (n (%))	21 (24.4)	308 (33.7)	0.080	21 (17.4)	234 (26.4)	**0.033**	99 (57.2)	578 (66.5)	**0.019**	141 (37.1)	1,120 (41.9)	0.074
Received food supplementation at last once (n (%))	3 (3.5)	42 (4.6)	0.636	0	18 (2.0)	0.114	6 (3.5)	22 (2.5)	0.487	9 (2.4)	82 (3.1)	0.452
At least one ultrasound (n (%))	73 (84.9)	803 (87.9)	0.424	53 (43.8)	478 (53.8)	**0.038**	64 (37.0)	418 (48.1)	**0.007**	190 (50.0)	1,699 (63.6)	**<0.001**
Continues to take iron and folic acid (adherence at least once during FU) (n (%))	59 (68.6)	676 (74.0)	0.282	64 (52.9)	580 (65.3)	**0.008**	101 (58.4)	598 (68.8)	**0.008**	224 (59.0)	1,854 (69.4)	**<0.001**
Counselling on postpartum family planning at least once during FU ANC (n (%))	0	40 (4.4)	**0.048**	11 (9.1)	153 (17.2)	**0.023**	67 (38.7)	396 (45.6)	0.098	78 (20.5)	589 (22.1)	0.501
**DELIVERY CARE (among women who gave birth in facilities)**	**N = 64**	**N = 743**		**N = 95**	**N = 733**		**N = 138**	**N = 665**		**N = 297**	**N = 2,141**	
**Consent and privacy for vaginal examinations** (n (%))												
Vaginal examination	29 (45.3)	316 (42.5)	0.666	71 (74.7)	491 (67.0)	0.128	109 (79.0)	515 (77.4)	0.692	209 (70.4)	1,322 (61.8)	**0.004**
Vaginal examination with permission	12 (18.8)	153 (20.6)	0.726	54 (56.8)	383 (52.3)	0.399	78 (56.5)	440 (66.2)	**0.031**	144 (48.5)	976 (45.6)	0.348
Privacy during vaginal examination	20 (31.3)	234 (31.5)	0.968	66 (69.5)	464 (63.3)	0.238	102 (73.9)	490 (73.7)	0.956	118 (63.3)	1,188 (55.5)	**0.011**
**Appropriate pain relief**												
Requested for pain relief	8 (12.5)	159 (21.4)	0.092	21 (22.1)	228 (31.1)	0.072	27 (19.6)	152 (22.9)	0.398	56 (18.9)	539 (25.2)	**0.017**
Offered/received pain relief	13 (20.3)	254 (34.2)	**0.024**	43 (45.3)	511 (69.7)	**<0.001**	121 (87.7)	594 (89.3)	0.574	177 (59.6)	1,359 (63.5)	0.194
Privacy during delivery	35 (57.8)	445 (61.9)	0.492	87 (91.6)	677 (92.4)	0.593	105 (77.8)	561 (86.3)	**0.012**	227 (76.4)	1,682 (78.6)	0.672
**IMMEDIATE POSTPARTUM CARE (among women who gave birth in facilities)**	**N = 59**	**N = 698**		**N = 94**	**N = 728**		**N = 138**	**N = 658**		**N = 291**	**N = 2,084**	
Postnatal check-up before discharge	21 (35.6)	412 (59.0)	**<0.001**	87 (92.6)	672 (92.3)	0.933	119 (86.2)	602 (91.5)	0.055	227 (78.0)	1,686 (80.9)	0.242
**Immediate postnatal counselling**	**N = 61**	**N = 704**	0.108	**N = 92**	**N = 719**	**0.045**	**N = 137**	**N = 649**		**N = 290**	**N = 2,070**	
What the baby should eat	17 (27.9)	269 (38.3)	0.144	74 (80.4)	631 (87.9)	**0.004**	92 (67.2)	495 (76.4)	**0.024**	183 (63.1)	1,395 (67.4)	0.143
Umbilical cord care	16 (26.2)	250 (35.5)	**0.003**	79 (85.9)	675 (94.0)	**0.018**	100 (73.0)	488 (75.4)	0.673	195 (67.2)	1,413 (68.3)	0.719
Keep baby warm	7 (11.5)	208 (29.6)	0.313	72 (78.3)	626 (87.3)	0.083	84 (61.3)	441 (68.0)	0.550	163 (56.2)	1,275 (61.7)	0.075
Return for baby vaccinations	35 (57.4)	449 (63.9)	0.292	85 (92.4)	691 (96.2)	0.530	123 (89.8)	599 (92.3)	0.328	243 (83.8)	1,739 (84.0)	0.925
Hand washing with soap	12 (19.7)	181 (25.8)	0.389	73 (79.4)	589 (82.0)	0.075	93 (67.9)	464 (71.5)	0.398	178 (61.4)	1,234 (59.4)	0.572
Danger signs for the baby	13 (21.3)	185 (26.4)	0.325	59 (64.1)	524 (73.0)		77 (56.2)	419 (64.7)	0.062	149 (51.4)	1,128 (54.6)	0.311
Danger signs for the mother	14 (23.0)	203 (28.9)	**0.041**	55 (59.8)	510 (70.9)	**0.028**	77 (56.2)	408 (63.2)	0.128	146 (50.3)	1,121 (54.2)	0.301
Immediate postpartum counselling completeness index (mean (SD))	26.7 (28.4)	35.5 (32.5)		77.2 (29.01)	84.5 (23.5)	**0.006**	67.4 (29.9)	73.1 (30.1)	**0.043**	61.9 (34.7)	64.2 (35.8)	0.301
**USER EXPERIENCE**												
**First ANC visit**	**N = 86**	**N = 914**		**N = 121**	**N = 888**		**N = 173**	**N = 869**		**N = 380**	**N = 2,671**	
Rates overall quality of first ANC visit as very good/excellent	49 (57.0)	487 (53.3)	0.511	72 (59.5)	560 (63.1)	0.448	84 (48.6)	485 (55.8)	0.080	205 (54.0)	1,532 (57.4)	0.209
**Delivery care**	**N = 61**	**N = 715**		**N = 94**	**N = 727**		**N = 138**	**N = 665**		**N = 293**	**N = 2,107**	
Rates overall quality of delivery care as very good/excellent	31 (50.8)	308 (43.1)	0.242	43 (45.7)	380 (52.3)	0.234	64 (46.4)	346 (52.0)	0.227	138 (47.1)	1,034 (49.1)	0.526
Very likely to recommend the provider for childbirth	41 (68.3)	391 (55.2)	**0.048**	55 (58.5)	458 (63.0)	0.398	78 (56.5)	393 (59.6)	0.511	174 (59.6)	1,242 (59.3)	0.914

*Quality indicators are described in Box 1.

**Fig 1:**
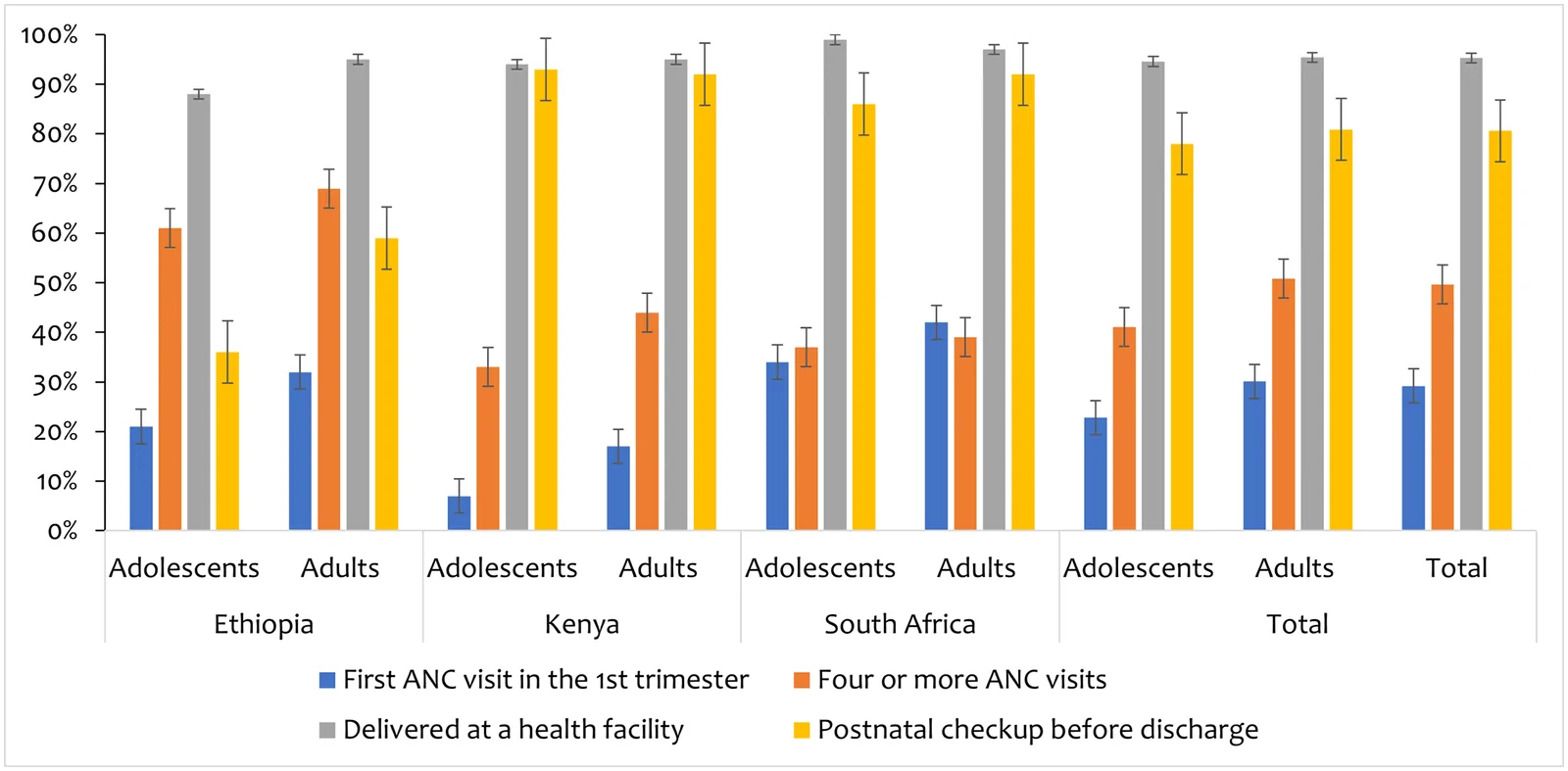
Continuity of maternal healthcare between adolescents and adults enrolled in the MNH eCohort study in Ethiopia, Kenya and South Africa, 2023-2024.

Adolescents experienced lower quality and less complete maternal health care across the antenatal, delivery, and postnatal continuum compared to adult women. They were less likely to initiate ANC early, attend the recommended number of visits, or receive essential interventions such as ultrasounds, urine tests, and IFAS. Counselling and postnatal care (PNC) were also less comprehensive among adolescents, contributing to lower continuity of care scores. However, adolescents were more likely to receive the recommended screenings and treatments during the first ANC visit.

Regarding delivery outcomes, adolescents were more likely to experience mistreatment during delivery and underwent significantly more episiotomies. In contrast, adult women had higher caesarean section rates. Country-specific patterns varied. In Ethiopia, adolescents had lower facility delivery and postnatal check-up rates and experienced more mistreatment than adults. In Kenya, important differences were observed in ANC services and access to pain relief. In South Africa, adolescents experienced more mistreatment and obstetric complications. Across all three countries, episiotomy was substantially more common among adolescents than adults. More detailed results are presented in the subsections below.

#### Antenatal care.

Less than one-third of women attended their first ANC visit during the first trimester, with significantly higher rates among adults (30%) compared to adolescents (22%, p = 0.003) (Fig 1). Adolescents received more comprehensive screenings and treatments during the first ANC visit than adults (72.6% vs. 68.0%, p < 0.001). However, first ANC counselling completeness was low, averaging about 50% (Table 2). With these variations in service delivery, just over half of the women rated the quality of their first ANC visit as very good or excellent, with no significant differences between adolescents and adults across all countries (Table 2).

Adolescents attended fewer follow-up (FU) ANC visits than adults (2.0 vs. 2.5, p < 0.001). Less than half of all women received additional blood (48%) or urine tests (41%) during FU visits. However, adolescents were significantly less likely than adults to receive urine tests in Kenya (17.4% vs. 26.4%, p = 0.033) and South Africa (57.2% vs. 66.5%, p = 0.019) (Table 2).

Over two-thirds of all women received at least one ultrasound during ANC, with adolescents less likely than adults to undergo the procedure (63.6% vs. 50.0%, p < 0.001). While ultrasound coverage was high in Ethiopia, where over 80% of both groups received at least one scan, adolescents were significantly less likely than adults to receive ultrasounds in Kenya (43.8% vs. 53.8%, p = 0.038) and South Africa (37.0% vs. 48.1%, p = 0.007). In contrast, food supplementation during ANC visits was rare, with only 3% of adult versus 2% adolescent women reporting to have received it. Notably, no Kenyan adolescents (0/121) received food supplementation during the study period (Table 2).

Adherence to IFAS during FU ANC visits was 68% overall, with significantly lower adherence among adolescents compared to adult women (59.0% vs. 69.4%, p < 0.001). This disparity was particularly pronounced in Kenya (52.9% vs. 65.3%, p = 0.008) and South Africa (58.4% vs. 68.8%, p = 0.008). However, adherence rates were relatively high in Ethiopia, where more than two-thirds of both groups continued IFAS use. Regarding postpartum family planning, only 22% of women received counselling at least once during FU ANC visits, with significantly lower rates among adolescents compared to adults in Ethiopia (0 vs. 4.4%, p = 0.048) and Kenya (9.1% vs. 17.2%, p = 0.023) (Table 2).

The average total number of ANC visits was four, ranging from three to five across all age groups and countries. However, adult women attended significantly more ANC visits than adolescents, particularly in Kenya and Ethiopia. Nearly half of all women attended more than four ANC visits, with adult women significantly more likely than adolescents to do so (51% vs. 41%, p < 0.001), with notable differences observed in Kenya (Table 2 and Fig 1).

#### Delivery care.

Over 95% of women delivered in a health facility, with facility-based delivery rates ranging from 88% to 99% across both age groups and the three countries. In Ethiopia, adolescents were less likely than adults to deliver in a health facility (88% vs. 95%, p = 0.016) (Fig 1).

More than 60% of women reported undergoing VEs, with higher rates among adolescents than adults (70.4% vs. 61.8%, p = 0.004). However, only 45% of women reported being asked for permission before the procedure. In Kenya, adolescents were less likely than adults to report being asked for consent (56.5% vs. 66.2%, p = 0.031). Additionally, just over half of the women indicated that privacy was maintained during VEs, with adolescents reporting significantly higher rates of privacy compared to adult women (63.3% vs. 55.5%, p = 0.011). In contrast, privacy during delivery was reported by over 78% of women. However, significant differences were noted in South Africa, where adolescents were less likely than adult women to report having privacy during delivery (77.8% vs. 86.3%, p = 0.012) (Table 2).

Regarding pain relief during delivery, only 24% of women requested for it, with a lower proportion among adolescents. Nevertheless, over 60% of women reported either receiving or being offered pain relief. Despite this, significant disparities existed, with adolescents having lower access to pain relief than adults in Ethiopia (20.3% vs. 34.2%, p = 0.024) and Kenya (45.3% vs. 69.7%, p < 0.001) (Table 2).

Less than half of the women (49%) rated the quality of delivery care as very good or excellent, with no significant differences between adolescents and adults. However, 59% of women indicated a high likelihood of recommending their childbirth provider to others. In Ethiopia, adolescents were significantly more likely than adults to recommend their provider (68.3% vs. 55.2%, p = 0.048) (Table 2).

#### Immediate postpartum care.

Among women who delivered in a health facility, 81% reported receiving a postnatal check-up before discharge. However, rates were significantly lower in Ethiopia, where just over one-third of adolescents and more than half of adults received a postnatal check-up (Table 2 and Fig 1).

On average, women received 64% of the recommended immediate postnatal counselling messages, with adult women receiving significantly more counselling than adolescents across all three countries. Adults were more likely to be counselled on key postnatal topics like infant feeding, umbilical cord care, keeping the baby warm, and recognising maternal danger signs, particularly in Kenya (Table 2).

The overall continuity of care index was 58%. Continuity was lower among adolescents than adults (53% vs. 59%, p < 0.001). This pattern was observed across all three countries, with significant differences in Ethiopia and Kenya (Fig 1).

### Experience of adverse delivery outcomes

Table 3 presents the proportion of adolescent and adult participants who experienced mistreatment or obstetric complications. Overall, 19.7% of women reported experiencing mistreatment, including physical or verbal abuse during delivery, with significantly higher rates among adolescents than adults (26.9% vs. 18.6%, p = 0.003) especially in Ethiopia and South Africa (Table 3).

**Table 3:** Prevalence of adverse delivery outcomes between adolescents and adults enrolled in the MNH eCohort study in Ethiopia, Kenya and South Africa, 2023-2024.

	Ethiopia			Kenya			South Africa			Total			
	Adolescents N = 64	Adults N = 743	P-value	Adolescents N = 95	Adults N = 733	P-value	Adolescents N = 138	Adults N = 665	P-value	Adolescents N = 297	Adults N = 2,141	P-value	Total N = 2,438
	n (%)	n (%)		n (%)	n (%)		n (%)	n (%)		n (%)	n (%)		n (%)
Mistreatment during delivery (physical/mental)	23 (39.0)	182 (26.0)	**0.030**	14 (14.7)	79 (10.8)	0.497	43 (31.4)	138 (21.1)	**0.009**	80 (26.9)	399 (18.6)	**0.003**	479 (19.7)
Caesarean section	7 (10.9)	158 (21.3)	0.135	16 (16.8)	222 (30.3)	**0.006**	42 (30.4)	246 (37.0)	0.238	65 (21.9)	526 (29.2)	**0.026**	691 (28.2)
Episiotomy	29 (45.3)	170 (22.9)	**<0.001**	44 (46.3)	160 (21.8)	**<0.001**	75 (54.4)	189 (28.4)	**<0.001**	148 (49.8)	519 (24.2)	**<0.001**	667 (27.4)
**Other obstetric complications**													
Prolonged labour (over 12 hours)	14 (23.4)	130 (17.5)	0.310	18 (18.0)	132 (18.0)	0.823	31 (22.5)	80 (12.0)	**0.001**	64 (21.6)	342 (16.0)	0.054	406 (16.7)
Admitted to ICU	3 (4.7)	9 (1.2)	0.076	1 (1.1)	12 (1.6)	0.666	5 (3.6)	13 (2.0)	0.228	9 (3.0)	34 (1.6)	0.201	43 (1.8)
Blood transfusion	1 (1.6)	12 (1.6)	0.579	2 (2.1)	26 (3.6)	0.464	15 (10.9)	37 (5.6)	**0.033**	18 (6.1)	75 (3.5)	0.093	93 (3.8)
Obstetric fistula	1 (1.6)	8 (1.1)	0.854	1 (1.1)	8 (1.1)	0.973	5 (3.6)	14 (2.1)	0.307	7 (2.4)	30 (1.4)	0.355	37 (1.5)
Self-reported severe health problems	3 (4.7)	89 (12.0)	**0.030**	1 (1.1)	49 (6.7)	**0.034**	9 (6.5)	51 (7.7)	0.329	13 (4.4)	189 (8.8)	**0.015**	202 (8.3)
At least 1 other obstetric complication ^a^	18 (28.1)	202 (27.2)	0.872	20 (21.1)	191 (26.1)	0.292	54 (39.1)	162 (24.3)	**<0.001**	92 (31.0)	255 (25.9)	0.065	647 (26.5)
Peripartum depression (PHQ2 > 3)	2 (3.1)	76 (10.2)	0.174	3 (3.2)	39 (5.3)	0.664	11 (8.0)	37 (5.6)	0.275	16 (5.4)	152 (7.1)	0.409	46 (1.9)

^a^Included the reporting of at least one of the other obstetric complications including prolonged labour (over 12 hours), admitted to ICU, blood transfusion, obstetric fistula or self-reported severe health problems

Approximately 28.2% of women underwent a caesarean section (CS), while 27.4% experienced an episiotomy. The CS rates were significantly higher among adult women (29.2% vs. 21.9%, p = 0.026), whereas episiotomies were higher among adolescents (49.8% vs. 24.2%, p < 0.001). Country-specific differences were observed, with CSs being more frequent among adults in Kenya, while episiotomy rates were significantly higher among adolescents across all three countries (Table 3).

In addition, 26.4% of women reported experiencing at least one obstetric complication (other than CSs or episiotomies) during delivery. In South Africa, adolescents had significantly higher rates of obstetric complications than adults (39.1% vs. 24.3%, p < 0.001). Prolonged labour (lasting more than 12 hours) was the most frequently reported complication (16.7%), while admission to the ICU was the least common (1.8%). Adolescents in South Africa were significantly more likely than adults to experience prolonged labour (22.5% vs. 12.o%, p = 0.001) and require blood transfusions (10.9% vs. 5.6%, p = 0.033). However, in Ethiopia and Kenya, adolescents reported significantly lower rates of severe health complications compared to adults (Table 3).

#### Factors associated with mistreatment during delivery among adolescents from Ethiopia, Kenya and South Africa.

Multivariable analysis identified several factors significantly associated with mistreatment during delivery among adolescents. Adolescents from Kenya had significantly lower odds of reporting mistreatment compared to those from Ethiopia (adjusted odds ratio [aOR]: 0.19, 95% confidence interval [CI]: 0.05–0.75, p = 0.018). Other protective factors included experiencing privacy during delivery (aOR: 0.44, 95% CI: 0.21–0.92, p = 0.030) and high-quality delivery care ratings (aOR: 0.37, 95% CI: 0.18–0.76, p = 0.007). In contrast, adolescents from rural areas (aOR: 2.61, 95% CI: 1.11–6.13, p = 0.028), those who delivered in government-operated facilities (aOR: 4.33, 95% CI: 1.44–13.1, p = 0.009), and those who had experienced IPV (aOR: 3.31, 95% CI: 1.16–9.39, p = 0.025) had significantly higher odds of mistreatment (Table 4).

**Table 4:** Results of multivariable logistic regression models for the factors associated with mistreatment during delivery among adolescents enrolled in the MNH eCohort study in Ethiopia, Kenya and South Africa, 2023-2024 (N = 280).

Factors	Adjusted Odds Ratio (aOR)	95% CI	p-value
**Country**			
Ethiopia	Ref		
Kenya	**0.19**	**0.05–0.75**	**0.018**
South Africa	0.36	0.08–1.75	0.207
**Region**			
Urban	Ref		
Rural	**2.61**	**1.11–6.13**	**0.028**
**Facility ownership**			
FBO^a^/NGO^b^/Private	Ref		
Government	**4.33**	**1.44–13.1**	**0.009**
**Experienced Intimate Partner Violence (emotional/physical)**			
No	Ref		
Yes	**3.31**	**1.16–9.39**	**0.025**
**Privacy during delivery**			
No	Ref		
Yes	**0.44**	**0.21–0.92**	**0.030**
**User experience**			
**Overall rating of the quality of delivery care**			
Poor/fair/Good			
Excellent/Very good	**0.37**	**0.18–0.76**	**0.007**

^a^FBO-faith-based organization; ^b^NGO- non-governmental organization

#### Factors associated with obstetric complications among adolescents from Ethiopia, Kenya and South Africa.

The multivariable analysis identified three significant predictors of obstetric complications among adolescents. Adolescents residing in rural areas had more than twice the odds of experiencing at least one obstetric complication compared to those from urban areas (aOR: 2.25, 95% CI: 1.09–4.63, p = 0.028). Similarly, adolescents who experienced symptoms of antenatal depression were significantly more likely to report obstetric complications than those without depressive symptoms (aOR: 2.43, 95% CI: 1.19–5.00, p = 0.015). Lastly, adolescents who visited another health facility before reaching their final delivery facility had nearly double the odds of experiencing an obstetric complication during labour (aOR: 1.96, 95% CI: 1.02–3.80, p = 0.045) (Table 5).

**Table 5:** Results of multivariable logistic regression models for the factors associated with obstetric complications among adolescents enrolled in the MNH eCohort study in Ethiopia, Kenya and South Africa, 2023-2024 (N = 284).

Factors	Adjusted Odds Ratio (aOR)	95% CI	p-value
**Place of residence**			
Urban	Ref		
Rural	**2.25**	**1.09–4.63**	**0.028**
**Current medical/obstetric history**			
Experienced prenatal depression (PHQ9 > 5)			
No	Ref		
Yes	**2.43**	**1.19–5.00**	**0.015**
**Visited another facility before delivery facility**			
No	Ref		
Yes	**1.96**	**1.02–3.80**	**0.045**

## Discussion

This study examined the quality, continuity, and experiences of maternal healthcare among 380 pregnant adolescents and 2,671 pregnant adult women in Ethiopia, Kenya, and South Africa. We compared care across the maternal healthcare continuum and identified factors associated with mistreatment and obstetric complications among adolescents.

Adolescents received relatively comprehensive screening and treatment during their first ANC visit. However, this was not sustained across the maternal healthcare continuum, with adolescents experiencing poorer continuity of care overall. Early initiation of ANC was significantly lower among adolescents. Barriers such as stigma, limited knowledge, transport and financial constraints, unplanned pregnancies and fear of provider judgment may contribute to delayed or discontinuous care [17,18,37,38]. Improving early ANC uptake and continuity of care among adolescents requires adolescent-friendly outreach, early pregnancy identification strategies, and community-level interventions to reduce stigma and improve access. Evidence from SSA suggests that adolescent-friendly approaches combining healthcare provider training, community outreach and health education, youth engagement, and adaptations to make facilities more responsive to adolescents’ needs can facilitate access to and uptake, although effectiveness varies across settings [39,40].

Even when adolescents accessed ANC services, they were less likely to receive comprehensive counselling and essential services such as IFAS, ultrasound, and postpartum family planning. Only half of all the women received complete counselling during their first ANC visit. Provider bias, poor communication, rushed consultations or inadequate training may contribute to these gaps [41]. These quality gaps undermine adolescents’ ability to make informed decisions, weakening birth preparedness and future care-seeking. Strengthening adolescent-focused provider training, supervision, and integrated counselling protocols are necessary to close these gaps.

During the intrapartum period, facility delivery rates were high overall, in contrast to the rates reported across other countries [42,43]. This may partly reflect the facility-based recruitment of women attending their first ANC visit, as ANC attendance is associated with subsequent facility-based delivery [44]. However, adolescents in Ethiopia were less likely than adults to deliver in facilities. Barriers such as stigma, fear of healthcare providers, financial constraints, lack of support or preference for traditional birth settings may contribute to this difference [13,17,45,46]. This underscores the need for adolescent-responsive interventions to reduce persistent barriers to facility-based delivery.

Adolescents also reported poorer experiences of childbirth, including inadequate consent for VEs and pain relief. These findings are consistent with previous reports of adolescents being more vulnerable to disrespect and abuse during childbirth potentially due to power imbalances and provider bias [47–49]. This highlights the urgent need to institutionalise respectful maternity care standards, ensure provider accountability and prioritise adolescent-specific safeguards.

Postnatal care was similarly inadequate, particularly in Ethiopia, where only one-third of adolescents received a postnatal check-up before discharge. Adolescents also received less counselling on key topics such as infant feeding, umbilical cord care, thermal care for newborns and maternal danger signs. Given that the immediate postpartum period is critical for detecting life-threatening complications [50], these gaps are concerning. The particularly low levels of PNC in Ethiopia may reflect limited awareness, distance and access barriers, culture and lower use of maternal health services [17,51,52]. Health systems must improve postnatal outreach, education, and follow-up for adolescent mothers, particularly in low-resource settings. Given the self-reported nature of the data, these findings also underscore the need for counselling to be tailored to adolescents’ levels of understanding.

Mistreatment during childbirth was reported by one in five women, with higher rates among adolescents. This aligns with evidence of age-related discrimination in maternity care contributing to negative care experiences and discouraging future service use [20,47,48,53]. These findings highlight the need to strengthen respectful care through provider accountability, community engagement and adolescent empowerment.

Obstetric complications were more frequent among adolescents, particularly prolonged labour and the need for blood transfusion. Adolescents were also more likely to undergo episiotomy, a potentially harmful practice when performed routinely. This may be partly explained by their biological immaturity, such as incomplete pelvic development and reduced uterine efficiency [54,55]. High rates of blood transfusion suggest higher incidences of postpartum haemorrhage, often exacerbated by prolonged labour. These findings align with evidence linking adolescent pregnancies to higher risks of obstetric complications in resource-limited settings [2]. However, delayed ANC and poor continuity of care may also contribute to worse outcomes. These findings highlight the need for early identification of risks and improved continuity and quality of maternal care for adolescents throughout pregnancy and childbirth.

Risk factor analysis highlighted several vulnerabilities among adolescents. Rural residence was associated with adverse outcomes. This may reflect barriers such as long distances to facilities, provider shortages, and limited privacy [47,49]. Adolescents who visited another facility before reaching their final delivery facility also had higher odds of obstetric complications. This may reflect poor referral practices, and inadequate communication and coordination between facilities [56–58]. However, this may also reflect referral of adolescents who were already experiencing complications. Antenatal depression and IPV were also associated with adverse outcomes and may affect care-seeking and increase physiological stress [59,60]. These findings call for integrated, multisectoral interventions that strengthen referral systems and incorporate mental health and IPV screening and psychological support into routine ANC.

Privacy and perceived high-quality of delivery care were protective factors against mistreatment, suggesting that positive experiences can improve trust and engagement. However, these perceptions may also reflect broader facility-level quality where more patient-centred care is routinely provided. Being in Kenya was also protective, possibly reflecting national efforts to promote respectful maternity care [29]. These findings reinforce the importance of dignity, privacy and person-centred maternal care [61].

Adolescents rated care similarly to adults despite receiving poorer care, possibly reflecting lower expectations or limited prior exposure to health services. This discrepancy underscores the need to include objective quality metrics in adolescent care evaluations and not rely solely on patient satisfaction. More broadly, country-level differences should be interpreted in the context of varying healthcare systems and service delivery models across the three countries, which may influence access, quality, and continuity of care.

This study has some limitations. Data were self-reported potentially introducing recall and social desirability bias. Small sample sizes in certain subgroups may limit the generalizability of the findings across diverse geographical and cultural contexts. The continuity-of-care index used a threshold of four ANC visits rather than the current WHO recommendation of eight contacts, potentially overestimating continuity. Facility-level clustering could not be accounted for because delivery facility identifiers were unavailable, which may have underestimated standard errors and increased the risk of Type I error. We also did not apply a formal strategy to identify confounders, limiting causal interpretation. Additionally, we did not stratify results by marital status or pregnancy intention, nor could we fully distinguish between demand- and access-side barriers with the available data. Participant retention from enrolment to postpartum follow-up ranged from 81% in South Africa to 89% in Kenya, potentially introducing selection bias. Lastly, the focus on three countries limits the broader applicability of the findings to other LMICs with different healthcare settings. Future studies should incorporate more robust data on social context and health system factors to better interpret disparities and guide targeted interventions.

## Conclusion

In conclusion, this study identified critical gaps in the quality and continuity of maternal healthcare among adolescents compared to adults in Ethiopia, Kenya, and South Africa. Adolescents initiated ANC later, attended fewer visits, or received less complete care across the maternal health continuum. They also experienced more mistreatment during childbirth and had an increased risk of obstetric complications, particularly those living in rural areas or facing antenatal depression, and IPV.

These findings reflect both demand-side challenges, like stigma and delayed pregnancy recognition, and potential supply-side barriers, including provider bias and insufficient adolescent-responsive care. Multi-faceted approaches are needed to strengthen continuity of care and institutionalise respectful, adolescent responsive maternity care. Adolescent-friendly services remain essential, as they respond to the unique developmental, social, and communication needs of younger patients. Health systems should prioritize integration of adolescent-responsive care into primary healthcare facilities through provider training in non-discriminatory and developmentally appropriate communication, the establishment of youth-dedicated clinic hours, and the use of peer navigators. Embedding mental health and IPV support into routine ANC is also essential, particularly given the heightened vulnerability of this population. Structural investments are also needed to strengthen rural health infrastructure and improve continuity of care through well-functioning referral systems. Together, these interventions can promote equity, improve health outcomes, and ensure dignified care for adolescents. Future research should investigate the influence of social norms, system responsiveness, and lived experiences of adolescents on care-seeking and outcomes to inform context-specific and adolescent-centred strategies to improve maternal health outcomes and promote equitable access to quality care.

## Supporting information

S1 Table Survey questions to assess mistreatment during facility-based childbirth in MNH Cohort Study. (PDF)

S2 Table Questions used to assess health literacy. (PDF)

S1 Checklist Inclusivity in Global Research Questionnaire. (DOCX)
