# Supplementary material for: Maternal healthcare quality, continuity, and experiences among pregnant adolescents and adult women in Ethiopia, Kenya, and South Africa: A cohort study
Source: PLOS Glob Public Health. 2026 Oct 5;6(10):e0004729. doi: 10.1371/journal.pgph.0004729 (PMC13637932; doi:10.1371/journal.pgph.0004729)
Supplement: Supplementary file 1 [file pgph.0004729.s001.pdf]

S1 Table. Survey questions to assess mistreatment during facility-based childbirth in MNH Cohort Study

1. *Physical abuse;*
   a. Were you pinched by a health worker or other staff?
   b. Were you slapped by a health worker or other staff?
   c. Were you physically tied to the bed or held down to the bed forcefully by a health worker or other staff?
   d. You had forceful downward pressure placed on your abdomen before the baby came out?
2. *Verbal abuse;*
   a. Were you shouted or screamed at by a health worker or other staff?
   b. Were you scolded by a health worker or other staff?
   c. Did the health worker or other staff member make negative comments to you regarding your sexual activity?
   d. The health worker or other staff threatened that if you did not comply, you or your baby would have a poor outcome?
