# Supplementary material for: Maternal healthcare quality, continuity, and experiences among pregnant adolescents and adult women in Ethiopia, Kenya, and South Africa: A cohort study
Source: PLOS Glob Public Health. 2026 Oct 5;6(10):e0004729. doi: 10.1371/journal.pgph.0004729 (PMC13637932; doi:10.1371/journal.pgph.0004729)
Supplement: Supplementary file 2 [file pgph.0004729.s002.pdf]

S2 Table. Questions used to assess health literacy

**1**	Now I would like to talk about something else. Have you ever heard of an illness called HIV/AIDS?	1 Yes<br>0 No → GO TO 3
**2**	Do you think that people can get the HIV virus from mosquito bites?	1 Yes<br>0 No<br>99 Don't know
**3**	Have you ever heard of an illness called tuberculosis or TB?	1 Yes<br>0 No → GO TO 5
**4**	Do you think that TB can be treated using herbal or traditional medicine made from plants?	1 Yes<br>0 No<br>99 Don't know
**5**	When children have diarrhea, do you think that they should be given less to drink than usual, more to drink than usual, about the same or it doesn't matter?	1 Less than usual<br>2 More than usual<br>3 About the same<br>4 It doesn't matter<br>99 Don't know
**6**	Is smoke from a wood burning traditional stove good for health, harmful for health or do you think it doesn't really matter?	1 Good<br>2 Harmful<br>3 Doesn't matter

Adapted from the India Health and Human Development Survey (IHDS): Desai, S. and Vanneman, R., 2015. *India human development survey-ii (ihds-ii), 2011-12* (Vol. 31). Ann Arbor, MI: Inter-university Consortium for Political and Social Research.
